# Complementation of Essential Yeast GPI Mannosyltransferase Mutations Suggests a Novel Specificity for Certain *Trypanosoma* and *Plasmodium* PigB Proteins

**DOI:** 10.1371/journal.pone.0087673

**Published:** 2014-01-29

**Authors:** Leslie K. Cortes, John J. Scarcelli, Christopher H. Taron

**Affiliations:** New England Biolabs, Ipswich, Massachusetts, United States of America; Federal University of São Paulo, Brazil

## Abstract

The glycosylphosphatidylinositol (GPI) anchor is an essential glycolipid that tethers certain eukaryotic proteins to the cell surface. The core structure of the GPI anchor is remarkably well conserved across evolution and consists of NH_2_-CH_2_-CH_2_-PO_4_-6Manα1,2Manα1,6Manα1,4-GlcNα1,6-*myo*-inositol-PO_4_-lipid. The glycan portion of this structure may be modified with various side-branching sugars or other compounds that are heterogeneous and differ from organism to organism. One such modification is an α(1,2)-linked fourth mannose (Man-IV) that is side-branched to the third mannose (Man-III) of the trimannosyl core. In fungi and mammals, addition of Man-III and Man-IV occurs by two distinct Family 22 α(1,2)-mannosyltransferases, Gpi10/PigB and Smp3/PigZ, respectively. However, in the five protozoan parasite genomes we examined, no genes encoding Smp3/PigZ proteins were observed, despite reports of tetramannosyl-GPI structures (Man_4_-GPIs) being produced by some parasites. In this study, we tested the hypothesis that the Gpi10/PigB proteins produced by protozoan parasites have the ability to add both Man-III and Man-IV to GPI precursors. We used yeast genetics to test the *in vivo* specificity of Gpi10/PigB proteins from several *Plasmodium* and *Trypanosoma* species by examining their ability to restore viability to *Saccharomyces cerevisiae* strains harboring lethal defects in Man-III (*gpi10*Δ) or Man-IV (*smp3*Δ) addition to GPI precursor lipids. We demonstrate that genes encoding PigB enzymes from *T. cruzi*, *T. congolense* and *P. falciparum* are each capable of separately complementing essential *gpi10*Δ and *smp3*Δ mutations, while *PIGB* genes from *T. vivax* and *T. brucei* only complement *gpi10*Δ. Additionally, we show the ability of *T. cruzi PIGB* to robustly complement a *gpi10*Δ/*smp3*Δ double mutant. Our data suggest that certain *Plasmodium* and *Trypanosoma* PigB mannosyltransferases can transfer more than one mannose to GPI precursors *in vivo*, and suggest a novel biosynthetic mechanism by which Man_4_-GPIs may be synthesized in these organisms.

## Introduction

The glycosylphosphatidylinositol (GPI) anchor serves to attach many eukaryotic proteins to the cell surface. GPIs are essential glycolipids that are assembled stepwise on a phosphatidylinositol backbone within the endoplasmic reticulum (ER) and become attached to the carboxy-termini of certain secretory proteins prior to exiting the ER and trafficking to the cell surface via the secretory pathway. In mammals, GPI anchored proteins serve a variety of functions on the cell surface and GPI biosynthesis is essential for normal development [1]. In fungi, GPI biosynthesis is essential for viability as GPI anchored proteins are critical components of the yeast cell wall [2]. In protozoan parasites, GPIs are important in host-parasite interaction and also play a role in the pro-inflammatory immune response to parasitic infection [3], [4], [5].

**Figure 1:**
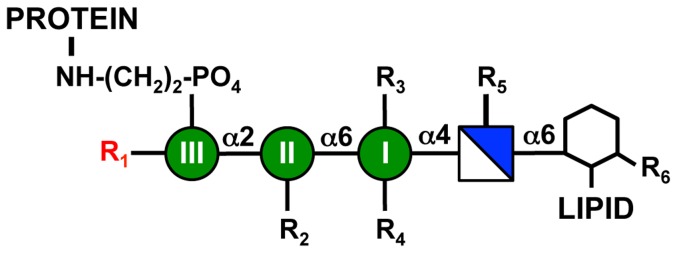
Schematic of the core GPI anchor glycan and its side chain modifications. GPI anchors consist of a highly conserved carbohydrate sequence that is linked to the inositol head group (hexagon) of phosphatidylinositol (hexagon + LIPID). The core GPI glycan contains glucosamine (blue/white square) and three mannoses (Man I-III, green circles). A protein is linked to the third mannose via an ethanolamine phosphate. R indicates positions where modification of the core GPI structure can occur, and possible R modifications in various species are described in Table 1. The red R_1_ indicates the position of fourth mannose addition.

Most known GPIs contain a common core structure of NH_2_-CH_2_-CH_2_-PO_4_-6Manα1,2Manα1,6Manα1,4-GlcNα1,6-*myo*-inositol-PO_4_-lipid (Figure 1) [6], [7], [8]. However, mature protein-bound GPI structure varies significantly across the whole of eukaryotic biology due to the presence of numerous moieties that become side branched to the GPI glycan and variations in lipid composition from organism to organism (Table 1) [2], [6], [7], [8], [9], [10]. Additionally, GPI structure is highly dynamic within any given organism with both additions and subtractions being made to the GPI molecule throughout its transport to the cell surface.

**Table 1:** Modifications of the GPI anchor core structure observed in various organisms^1^.

Organism	R1	R2	R3	R4	R5	R6	Lipid
*S. cerevisiae*	Man(α1,2)Man(α1,2) or Man(α1,3)Man(α1,2)	±EthNP	±EthNP	OH	OH	OH	DAG
*H. sapiens*	±Man(α1,2)	±EthNP	EthNP	(±NANA)(±Gal(β1,3))(±GalNAc(β1,4))	OH	±palmitate	AAG or DAG
*T. brucei*	OH	OH	OH	±Galα1−2(Galα1−2Galα1−6)Galα1−3	OH	OH	DMG
*T. cruzi*	Man(α1,2)	OH	OH	OH	AEP	OH	AAG/Cer
*P. falciparum*	Man(α1,2)	OH	OH	OH	OH	±myristic acid	DAG

Chart describing known side chain modifications of the GPI core in various species. R1-R6 and Lipid positions on the GPI core are indicated in Figure 1. DAG: diacylglycerol; AAG: alkylacylglycerol; DMG: dimyristylglycerol; Man: mannose; EthNP: ethanolamine phosphate; GalNAc: N-acetylgalactosamine; AEP: aminoethylphosphonate; Cer: ceramide.

1 See references 7, 10, 16, 17, 20

One interesting modification of the core GPI structure is the presence of a fourth mannose (Man-IV) α1,2-linked to the third mannose (Man-III) of the GPI. The Man-IV modification has been observed on GPIs isolated from various proteins in mammals [11], [12], [13], [14], fungi [15], and the protozoan parasites *Trypanosoma cruzi* [16] and *Plasmodium falciparum* [17]. The addition of Man-IV has been well defined in yeast and mammals [18], [19]. In both groups, Man-IV is added to GPI precursors during their biosynthesis by a conserved family of GPI mannosyltransferases (Smp3p/PigZp family). However, the two taxa differ in their relative requirement for Man-IV on the GPI glycan. In the yeast *Saccharomyces cerevisiae*, Man-IV is added by the essential Smp3 protein in a mandatory step of biosynthesis that precedes addition of EthN-P to Man-III and GPI transfer to protein [18]. Thus, it is believed that all yeast GPIs bear four mannoses. Conversely, in mammalian cells, trimannosyl-GPIs (Man_3_-GPIs) predominate with addition of Man-IV to GPIs by the PigZ protein occurring less frequently, perhaps in a tissue or cell-specific manner [19]. However, the physiological significance of this modification in mammals is still unclear.

The presence of Man-IV has also been observed in some protozoan parasites, but not others. For example, while the GPIs of *Trypanosoma brucei* do not contain Man-IV [20], the *P. falciparum* MSP-1 and MSP-2 proteins each bear GPIs with four mannoses [17]. Additionally, a glycoproteomic study of GPIs in *T. cruzi* showed that Man-IV was present on each of over 90 observed GPI structures suggesting that this organism exclusively synthesizes Man_4_-GPIs [16]. Interestingly, the genomes of both *P. falciparum* and *T. cruzi* lack genes encoding obvious orthologs of the Smp3/PigZ fourth mannosyltransferase family [21], suggesting that Man-IV addition to GPIs in these organisms likely occurs via a different mechanism.

In the present study, we have explored a hypothesis that the PigB family of GPI mannosyltransferases (GPI-MT-III) that add an α1,2-linked third mannose to GPIs in certain protozoan parasites may also be capable of adding the α1,2-linked fourth mannose. We have used yeast genetics to explore the *in vivo* specificity of various parasite PigB GPI mannosyltransferases by determining their ability to restore viability to yeast mutants harboring lethal defects in either Man-III or Man-IV addition to GPI precursor lipids. We report that the GPI-MT-III (PigB) proteins from *T. cruzi*, *T. congolense* and *P. falciparum* are capable of complementing both *gpi10*Δ and *smp3*Δ mutations. Additionally, we show that expression of *T. cruzi PIGB* can restore viability of a yeast double mutant defective in both Man-III and Man-IV addition to GPI precursors. This study represents the first evidence that a single GPI mannosyltransferase is capable of performing multiple sugar additions to the GPI anchor and provides insight into a possible mechanism for Man_4_-GPI formation in certain protozoan parasites.

## Materials and Methods

### Sequence database searching

The algorithms BLASTP and TBLASTN [22], [23] were used with default settings to identify putative Smp3p and Gpi10p sequences in various eukaryotic genomes present in GenBank. Searches were performed using the BLAST server at the National Center for Biotechnology Information website (www.ncbi.nlm.nih.gov) with Smp3 and Gpi10 protein sequences from *S. cerevisiae* and mammals as search queries. In certain cases, *SMP3* or *GPI10* genes were deemed to be absent from a genome if no hits with an e-value less than 1×10^−2^ were observed using both BLASTP and TBLASTN and the genomic data searched represented at least 7X genome sequence coverage. All identified genes are listed in Table S1.

### Yeast strains and culture media

A haploid *S. cerevisiae* strain (YCT138) containing a lethal *smp3*::Kan^R^ null allele and the rescuing vector pGAL-*ScSMP3* (*URA3*, CEN) was previously described [19]. A similar haploid strain (YCT684) having a *gpi10*::Kan^R^ allele and a rescuing pGAL-*ScGPI10* vector (*URA3*, CEN) was constructed by introduction of pGAL-*ScGPI10* into a heterozygous *GPI10*/*gpi10*::Kan^R^ diploid (Life Technologies) and dissection of tetrads to isolate a viable *gpi10*::Kan^R^ haploid segregant harboring pGAL-*ScGPI10*. These haploid stains (YCT138 and YCT684) were used as the base strains for the transformation of pPGK-*PIGB* expression plasmids and subsequent plasmid shuffling experiments.

To generate the *gpi10*Δ/*smp3*Δ double mutant strain, a haploid *gpi10*::Kan^R^ strain containing pGAL-*TcrPIGB* was mated with a haploid *smp3*::Kan^R^ strain containing pGAL-*TcrPIGB*. The resulting diploid was induced to sporulate and asci (“tetrads”) containing four haploid spores, the meiotic progeny of the cross, were dissected and assessed for their ability to grow on YPGal agar. For tetrads yielding four viable progeny, each haploid was scored for its resistance to kanamycin (G418). Tetrads showing the non-parental ditype segregation pattern were screened by PCR to identify haploids that contained both null *gpi10* and *smp3* loci as well as the pGAL-T*crPIGB* plasmid.

Compositions of yeast growth media SGal, YPGal and SD have been previously described [19], [24]. Amino acids were routinely supplemented in growth media to complement strain auxotrophies as previously described [24] and Geneticin (G418, Gibco) was used at a final concentration of 200 µg/mL. SD 5FOA medium contained 0.1% (w/v) 5-fluoroorotic acid (Toronto Research Chemicals) and 50 µg uracil per mL of SD medium.

### Expression of *GPI10, SMP3* and *PIGB* genes

*S. cerevisiae GPI10*, *SMP3* and *T.cruzi GPI10* were cloned into the pMW20 (pGAL) expression vector using a previously described scheme [19]. The coding regions of *S. cerevisiae GPI10* and *SMP3* were each amplified by PCR from *S. cerevisiae* genomic DNA and cloned into the PstI-BamHI sites (for *GPI10*) or NotI-AscI sites (for *SMP3*) of the yeast expression vector pPGK-415. Vector pPGK-415 was constructed in pRS-415 [25], [26] (CEN, *LEU2*) and contained 500 bp of the *PGK1* promoter and 249 bp of the *PGK1* terminator cloned between the SacI-XhoI sites. Three unique sites (NotI, PacI and AscI) are situated between the promoter and terminator to facilitate cloning of cDNA sequences for expression. Wild-type cDNA sequences of the *T. brucei PIGB* (AB033824.1), *T. cruzi PIGB* (XM_801669.1), *T. vivax PIGB* (HE573026.1, locus tag TVY486_1005620), and *T. congolense PIGB* (HE575323.1, locus tag TCIL3000_10_4690) genes were each synthesized and cloned into pUC57 (Genscript). Additionally, *P. falciparum PIGB* (XM_001350101.1) was synthesized with codon optimization for expression in yeast (Genscript). Each gene was amplified using Phusion High-Fidelity DNA Polymerase (New England Biolabs) with primers containing a C-terminal HA tag sequence and NotI or AscI restriction sites at the 5′ or 3′ end of the cDNA, respectively (See Table S2 for primers used in cloning). Amplified DNA fragments were digested and cloned into the NotI-AscI sites of pPGK-415. Chimera 1, consisting of nucleotides 1-306 of *TbPIGB* (which includes the ‘long motif’ region) fused to nucleotides 457-1827 of *TcrPIGB*, was synthesized (Genscript) and subcloned into pPGK-415 as described above. Chimera 2 was generated by ‘PCR knitting’ using primer sets 1, 16 and 2, 15 (Table S2) to first amplify fragments of *TcrPIGB* from a pPGK-415-*TcrPIGB* template. These fragments contained complementary overlapping regions corresponding to the *TbPIGB* ‘short motif’ region, and were fused together by a second round of PCR using primers 1 and 2. The resulting DNA fragment (chimera 2) contained the *TcrPIGB* cDNA with its short motif replaced with that of *TbPIGB* (i.e. a fusion consisting of *TcrPIGB* nt 1-1146, *TbPIGB* nt 1006-1053 and *TcrPIGB* nt 1195-1827). Chimera 2 was cloned into the NotI-AscI sites of pPGK-415 for expression as described above. Chimera 3 was generated using the same scheme as for the generation of chimera 2, but using chimera 1 as the template for the initial PCRs. This chimera therefore consisted of a fusion of nucleotides 1-306 of *TbPIGB*, nucleotides 457-1146 of *TcrPIGB*, nucleotides 1006-1053 of *TbPIGB* and nucleotides 1195-1827 of *TcrPIGB*. Typical Phusion PCR conditions were as follows: 98°C for 3 min, 10 cycles of 98°C for 15 s, 54°C for 15 s and 72°C for 1 min 15 s (20–30 s per kb), followed by 25 cycles of 98°C for 15 s, 63°C for 15 s and 72°C for 1 min 15 s (20–30 s per kb), and completed by a final extension at 72°C for 5 min. All primers used for the generation of expression constructs are listed in Table S2. Vectors were introduced into YCT684 and YCT138 using lithium acetate as described in [27] with the carrier DNA and PEG/TE/LiOAc replaced by NEB Yeast Transformation Reagent (New England Biolabs).

### Complementation of *gpi10* or *smp3* null mutations

Complementation of *gpi10* or *smp3* lethal null mutations was assessed by ‘plasmid shuffling’. This was performed by streaking isolated colonies of strains transformed with a pPGK-*PIGB* expression vector from selective medium (SGal) to non-selective medium (YPGal) to permit plasmid loss. These strains were subsequently subjected to two rounds of counter-selection on SD 5FOA agar plates at 25°C for 3–7 days to identify strains capable of growing in the absence of either of the parental *URA3*-containing vectors (pGAL-*ScSMP3* or pGAL-*ScGPI10*). Following the second round of 5FOA counter-selection, viable strains were assessed for complete loss of vectors pGAL-*ScSMP3* or pGAL-*ScGPI10* by PCR using primers that anneal to *URA3* (Primers P1 and P2, Table S3) and amplify a diagnostic amplicon if either vector is still present. Strains were also tested for the presence of a null *gpi10* or *smp3* locus (Primers P3–P5, Table S3) and the pPGK plasmid (Primers P6–P14, Table S3). See below for DNA extraction and PCR reaction conditions. All plasmid shuffling experiments were repeated at least twice.

### PCR confirmation of yeast strain genotypes

Total DNA was extracted from yeast strains as previously described [28]. PCR was performed using the Phusion High Fidelity Master Mix with HF Buffer (New England Biolabs), the primers listed in Table S3 and 25 ng of isolated DNA. Typical PCR cycling conditions were 95°C for 5 min, followed by 35 cycles of 95°C for 15 s, 53°C for 15 s and 72°C for 20 s and a final 5 min extension at 72°C.

### Serial drop cultures of yeast strains

Serial dilutions of yeast cultures were used to assess the robustness of growth of engineered strains compared to control strains. Yeast cells were suspended in 5 mL of appropriate medium after which 1 mL was used to determine cell density via light scattering (OD_600_) in a spectrophotometer (Amersham). Cells in the remaining 4 mL were pelleted by centrifugation for 15 min at 1850×g and resuspended in appropriate medium to a concentration of 2×10^7^ cells per mL. Serial 10-fold dilutions were performed and 5 µL of each dilution was plated onto appropriate agar medium. Plates were incubated for 3–5 days at 25°C.

## Results

### Identification of GPI α(1,2)-mannosyltransferase families

Synthesis of the mannose core of the GPI glycan occurs via sequential action of three distinct GPI mannosyltransferases that transfer mannose from the polyisoprenoid sugar donor dolichol phosphomannose (Dol-P-Man) to the GPI precursor. These three enzymes (GPI-MT-I, GPI-MT-II and GPI-MT-III) add α(1,4)-, α(1,6)- and α(1,2)-linked mannose, respectively (For a review see reference [6]). The structures of known GPIs across eukaryotic biology almost invariably contain this arrangement of mannoses, and as such, these three protein families (Gpi14p/PigMp, Gpi18p/PigVp, and Gpi10p/PigBp, respectively) are each highly conserved and their genes are straightforward to identify in genomic sequences. It is worth noting that some apicomplexan parasites such as *Babesia bovis, Babesia microti*, *Theileria annulata*, and *Theileria parva* lack a Gpi10/PigB family protein and *B. bovis* has been further shown to contain GPI anchor structures containing only two mannoses [29], [30]. In mammals and yeast, a fourth mannose (Man-IV) that is α(1,2)-linked to Man-III may be added to varying degrees by the Dol-P-Man utilizing Smp3/PigZ mannosyltransferase family (GPI-MT-IV). While this enzyme functions separately from GPI-MT-III, the two protein families both belong to glycosyltransferase family 22 [31], harbor similar domain organization [32] and share some isolated protein sequence homology. To date, the only known mechanism for addition of Man-IV to GPIs is via the Smp3/PigZ family of mannosyltransferases.

Precise GPI structural data is available for a relatively small number of GPI anchored proteins from few classes of organisms. Therefore, to better understand what organisms might be able to add Man-III and/or Man-IV to the GPI core structure, we examined the distribution of the Gpi10/PigB and Smp3/PigZ protein families across a wide array of eukaryotic organisms (Table S1). The BLASTP algorithm was used to identify both Gpi10/PigB and Smp3/PigZ protein sequences in the NCBI database. Candidate Gpi10p/PigBp sequences were encoded by every genome we examined, consistent with this enzyme's essential role in formation of the conserved trimannosyl core GPI glycan. In contrast, Smp3p/PigZp sequences were not as widely present in animal genomes (Table S1). In vertebrates, probable Smp3/PigZ proteins were encoded by each of 46 mammalian genomes and 11 avian genomes, but were absent from all 6 fish genomes we searched. In invertebrates, putative Smp3/PigZ proteins were encoded by each of the 31 arthropod genomes we examined and were also found in several primitive multicellular animals. However, several genomes lacked Smp3/PigZ proteins including those of nematodes and sea urchins. Outside of Animalia, possible Smp3/PigZ proteins were found encoded by the genomes of most fungi, several green algae and phytoplankton, but were absent from each of the 10 plant genomes we analyzed.

Curiously, Smp3p/PigZp sequences were absent from the genomes of all protozoan parasites we examined (Table S1), despite prior observations that Man-IV is present on GPIs on certain proteins from *P. falciparum* [17] and is abundantly present on GPI structures from *T. cruzi* [3]. This suggested that these organisms might employ a different mechanism for addition of Man-IV to GPIs. We hypothesized that the GPI-MT-III (PigB) proteins from these organisms might be able to add two consecutive α(1,2)-linked mannoses (Man-III and Man-IV) to GPIs.

### Expression of parasite GPI mannosyltransferase genes in yeast *gpi* mutants

Yeast GPI biosynthesis involves the action of two independent α1,2-mannosyltransferases that transfer Man-III and Man-IV to the GPI precursor. The genes encoding these enzymes (*GPI10* and *SMP3*, respectively) are both essential for cell viability, and conditional mutants of each gene accumulate GPI lipid intermediates with glycan head groups no larger than two mannoses [33] or three mannoses [18], respectively. Furthermore, overexpressed Gpi10 and Smp3 proteins are not capable of substituting for each other *in vivo* [18]. The latter observation suggests that while both genes encode yeast α1,2-mannosyltransferases, each protein exhibits strict substrate specificity, with Gpi10p adding mannose to Man_2_-GPI precursors and Smp3p adding mannose to Man_3_-GPI precursors. Together, these prior observations support the interpretation that the Gpi10 and Smp3 proteins each perform distinct, mandatory mannose transfer events in the yeast GPI assembly pathway. In this study, we exploited this unique feature of yeast GPI synthesis to query the ability of various parasite *PIGB* mannosyltransferase genes to complement lethal *gpi10*Δ or *smp3*Δ mutations as a way to interrogate the specificity of PigB proteins *in vivo*.

**Figure 2:**
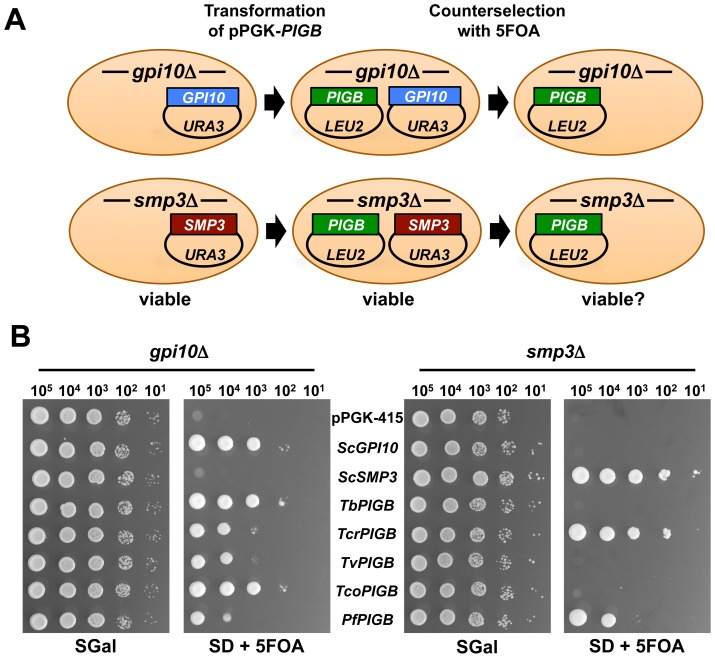
Complementation of yeast *gpi10* and *smp3* null mutants by protozoan *PIGB* genes. A. Schematic of yeast plasmid shuffling experiments. Haploid *gpi10*::Kan^R^ containing pGAL*-ScGPI10* (top row) or *smp3*::Kan^R^ containing pGAL*-ScSMP3* (bottom row) strains were transformed with protozoan pPGK*-PIGB* expression plasmids. Yeasts carrying both plasmids were then induced to lose one plasmid via growth on rich medium, and then strains containing only the pPGK plasmids were selected for by growth on 5FOA. B. Serial drop cultures of yeast on SGal and SD + 5FOA media. Note that all strains grow on SGal medium as they contain both the pGAL rescuing plasmid and the pPGK expression plasmid. Following selection on 5FOA only the strains containing *PIGB* genes that can complement the indicated null mutation are able to grow.

Complementation of yeast lethal *gpi10* and *smp3* null mutations by heterologously expressed parasite *PIGB* genes was assessed using a plasmid shuffling approach (Figure 2A). We used two haploid yeast strains that each contained a chromosomal deletion of either the *gpi10* or *smp3* gene and a rescuing *URA3*-containing plasmid that directed expression of wild-type *S. cerevisiae GPI10* or *SMP3* from the yeast *GAL10* promoter (strains YCT684 and YCT138, respectively). A second expression vector (pPGK) was used to express candidate parasite *PIGB* genes from *T. brucei* (*TbPIGB*), *T. cruzi* (*TcrPIGB*), *T. congolense* (*TcoPIGB*), *T. vivax* (*TvPIGB*) and *P. falciparum* (*PfPIGB*) via the *PGK1* promoter.

To assess the ability of protozoan *PIGB* genes to complement either *gpi10*Δ or *smp3*Δ, each pPGK-*PIGB* expression vector was separately introduced into *S. cerevisiae* YCT684 and YCT138. Plasmid shuffling was performed through counterselection for the loss of the *URA3*-containing vector by growth on medium containing 5FOA (Figure 2A). Each candidate protozoan *PIGB* gene permitted growth of *S. cerevisiae* harboring the lethal *gpi10*Δ mutation on 5FOA-containing medium (Figure 2B), indicating that each of the protozoan *PIGB* genes selected for this study was functional in yeast and was capable of substituting for the yeast GPI-MT-III *in vivo*. Furthermore, in the *smp3*Δ background, *TcrPIGB* and *PfPIGB* expression permitted robust growth on 5FOA-containing medium (Figure 2B). *TcoPIGB* in the *smp3*Δ background also demonstrated reproducible growth on 5FOA-containing medium, although its growth was significantly slower than other strains and was just above the pPGK-415 negative control at the 10^5^ cell dilution (Figure 2B, far right panel). Thus, the *Tcr*PigB, *Tco*PigB and *Pf*PigB proteins were able to overcome a GPI-MT-IV deficiency *in vivo*.

### Verification of viable strains

**Figure 3:**
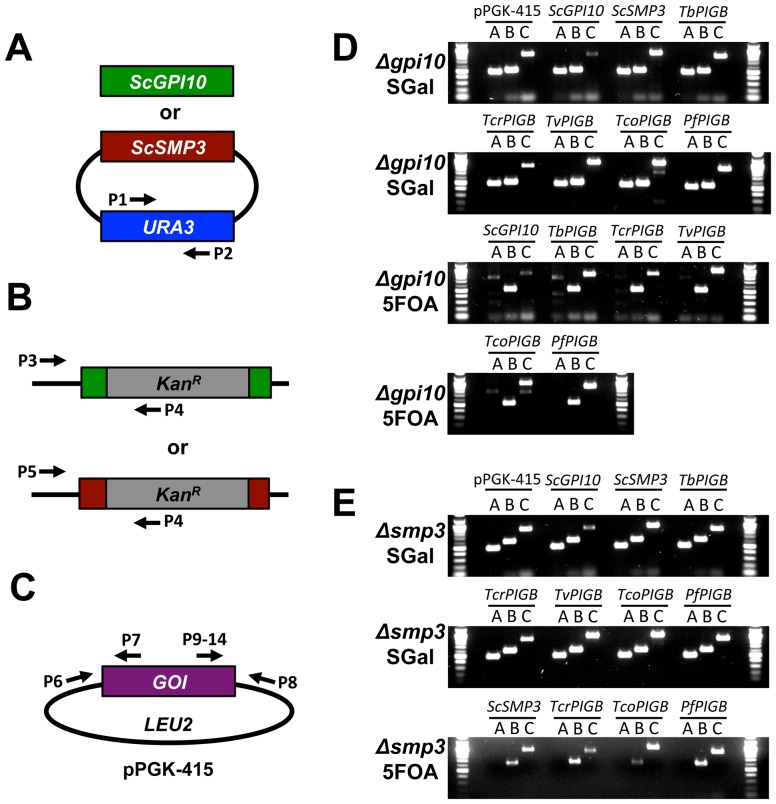
PCR confirmation of yeast strains. A–C. Schematics of PCR reactions used to confirm strains. See Table S3 for sequences of the indicated primers. A. To confirm the presence (on SGal) or absence (on 5FOA) of the pGAL rescuing plasmid, the *URA3* locus within this plasmid was amplified. B. To confirm that the endogenous *gpi10* or *smp3* locus was indeed null in all strains, forward primers residing in the 5′UTR of the genes were coupled with a reverse primer that sits within the kanamycin cassette that was inserted into the gene locus upon null strain generation. C. To confirm the presence of the pPGK plasmid, PCR was performed using a primer within the pPGK vector, coupled with a primer in the 3′end of the coding region of the *PIGB* gene of interest. D–E. Results of PCR experiments to confirm all yeast strains shown in Figure 2. DNA was isolated from all strains and the PCRs described in A–C were performed. D. *gpi10*Δ strains before (SGal) and after (5FOA) plasmid shuffling. E. *smp3*Δ strains before (SGal) and after (5FOA) plasmid shuffling. Reactions A–C correspond to the schematics A–C at left. Amplicon sizes are: Reaction A: 500 bp; Reaction B: 534 bp for *gpi10*, 616 bp for *smp3*; Reaction C: 902 bp for pPGK-415, 891 bp for pPGK-*ScGPI10*, 922 bp for pPGK-*ScSMP3*, 800 bp for pPGK-*TbPIGB*, 820 bp for pPGK-*TcrPIGB*, 879 bp for pPGK-*TvPIGB*, 932 bp for pPGK-*TcoPIGB* and 908 bp for pPGK-*PfPIGB.*

To verify complementation of the lethal *gpi10*Δ and *smp3*Δ mutations by expression of protozoan *PIGB* genes, we isolated genomic DNA from all strains both before and after plasmid shuffling and performed a series of diagnostic PCR tests to confirm strain genotypes and the presence or absence of pGAL- and pPGK-derived plasmids (see Figure 3A–C for schematic). PCR with primers specific for *URA3* was used to confirm the presence or absence of pGAL-*SMP3* or pGAL-*GPI10* in strains before and after plasmid shuffling (Figure 3A). PCR was then used to verify the presence of chromosomal *gpi10*::Kan^R^ or *smp3*::Kan^R^ alleles in the *gpi10*Δ or *smp3*Δ backgrounds, respectively (Figure 3B). Another PCR reaction was performed to confirm the presence of a pPGK-*PIGB* expression vector using individual forward primers that anneal to the 3′ end of each *PIGB* gene and a reverse primer specific for the pPGK backbone (Figure 3C).

We confirmed that all strains shown in Figure 2B contained the expected arrangements of plasmids and knockout loci (Figure 3D and E). In particular, the *URA3*-plasmid containing the wild-type *ScGPI10* or *ScSMP3* complementing gene was present in all strains prior to plasmid shuffling (Figure 3D), but had been lost in all surviving strains after 5FOA counterselection (Figure 3E). Of note, even though the *TcoPIGB* expressing strain had shown minimal growth on 5FOA-containing medium in the drop culture experiment, we were able to isolate DNA and confirm that it harbored an *smp3*::Kan^R^ locus, lacked the wild-type *S. cerevisiae SMP3* rescuing plasmid, and contained the pPGK-*TcoPIGB* plasmid (Figure 3E). From these data, we show that *TcrPIGB* and *PfPIGB*, and to a lessor extent *TcoPIGB*, are able to compensate for deficiencies in both Man-III and Man-IV addition to yeast GPI precursors *in vivo*.

### Complementation of a *gpi10*/*smp3* double null mutant

We sought further genetic evidence that some parasite *PIGB* genes are capable of performing the dual role of Man-III and Man-IV addition to GPI anchors. In a second complementation experiment, we investigated if *TcrPIGB* could restore viability to a haploid *gpi10*::Kan^R^/*smp3*::Kan^R^ double mutant. We elected to use *TcrPIGB* expression for this experiment because it showed the most robust complementation of *smp3*Δ.

**Figure 4:**
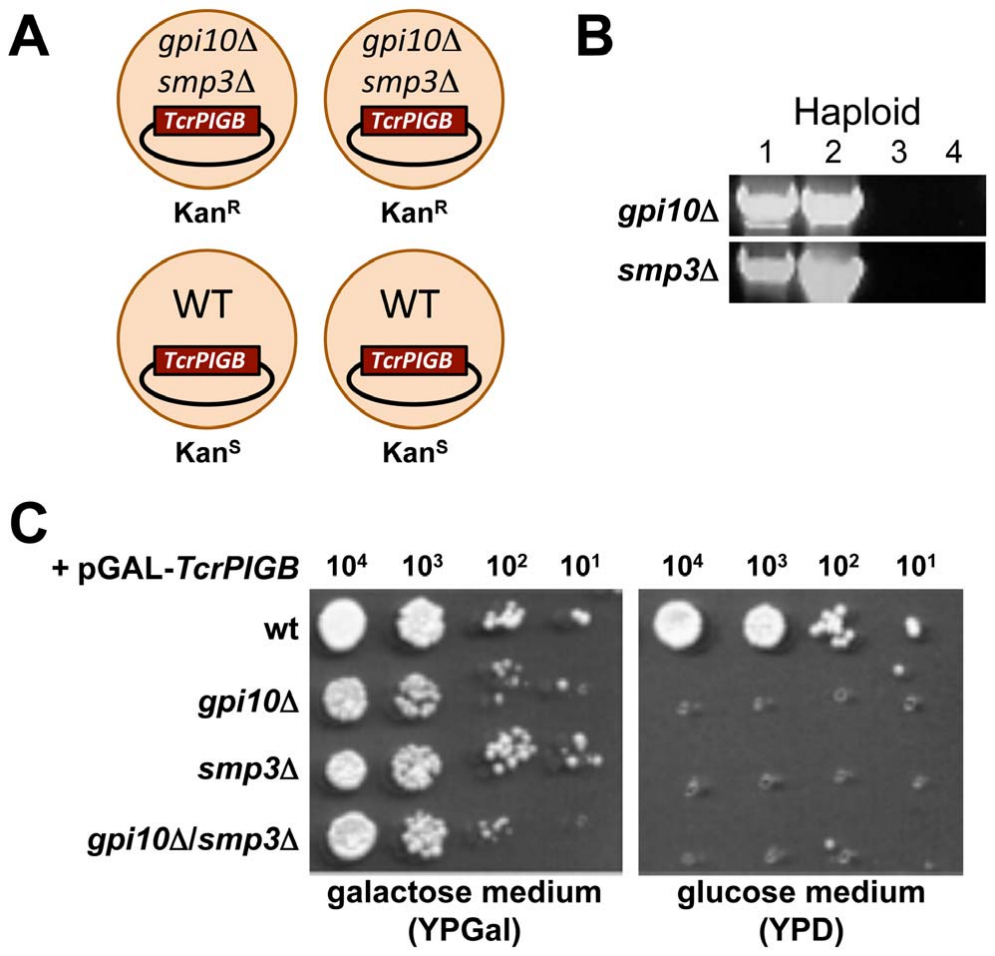
*T. cruzi PIGB* can complement a yeast strain deficient in both *gpi10* and *smp3*. A. Schematic of a tetrad showing the desired non-parental ditype pattern of kanamycin marker segregation generated from the mating of a haploid *gpi10*::Kan^R^ strain containing pGAL-*TcrPIGB* with a haploid *smp3*::Kan^R^ strain containing pGAL-*TcrPIGB*. B. PCR was used to test each haploid meiotic segregant from a non-parental ditype tetrad for the presence of *gpi10*Δ and *smp3*Δ alleles (see Figure 3B for PCR schematic). Haploids 1 and 2 are each double mutants that contain both null alleles. C. Serial drop cultures of both single *gpi10*Δ and *smp3*Δ mutant strains as well as the double mutant *gpi10*Δ*/smp3*Δ strain. Note that the strains grow on the galactose-containing medium that induces pGAL-*TcrPIGB* expression but not on the glucose medium, which represses pGAL-*TcrPIGB* expression.

We generated a double knockout strain by mating a haploid *gpi10*Δ strain containing pGAL-*TcrPIGB* with a haploid *smp3*Δ strain containing pGAL-*TcrPIGB* and inducing sporulation. Following dissection of ascospores representing the meiotic progeny of the cross, we readily obtained tetrads showing a non-parental ditype pattern of kanamycin marker segregation, with two Kan^R^ segregants (double mutants) and two Kan^S^ segregants (wild-type) (Figure 4A). PCR was used to show that the two viable Kan^R^ segregants each harbored both the *gpi10*::Kan^R^ and *smp3*::Kan^R^ alleles (Figure 4B). Finally, a representative viable double null strain was scored for its ability to grow on medium containing galactose or glucose to induce or repress *TcrPIGB* expression, respectively (Figure 4C). The double mutant grew robustly in the presence of galactose, but not in glucose, further indicating that its viability was entirely dependent on expression of *TcrPIGB* from the complementing vector pGAL-*TcrPIGB*.

These data unambiguously demonstrate that *TcrPIGB* is capable of restoring viability to cells defective in both Man-III and Man-IV addition to GPI precursors. The likeliest interpretation of these observations is that *Tcr*PigB is capable of efficiently adding both the third and fourth mannoses to yeast GPI precursors during their biosynthesis in the ER.

### Use of chimeric constructs to evaluate *TcrPIGB* substrate recognition *in vivo*

Little is known about the structure and function of Gpi10p/PigBp α(1,2)-mannosyltransferases. Several factors have precluded the use of classic *in vitro* biochemical characterization of these enzymes. First, members of this protein family are multi-spanning transmembrane proteins and are difficult to abundantly express and purify. Second, these enzymes transfer mannose from Dol-P-Man to low abundance intermediate lipids that cannot be purified in sufficient quantities for *in vitro* transferase assays. Therefore, we elected to use our system of complementation of yeast *gpi10*Δ and *smp3*Δ mutations to explore any relationship between highly conserved domains in Gpi10/PigB proteins and their ability to transfer Man-III or Man-IV to GPI precursors.

**Figure 5:**
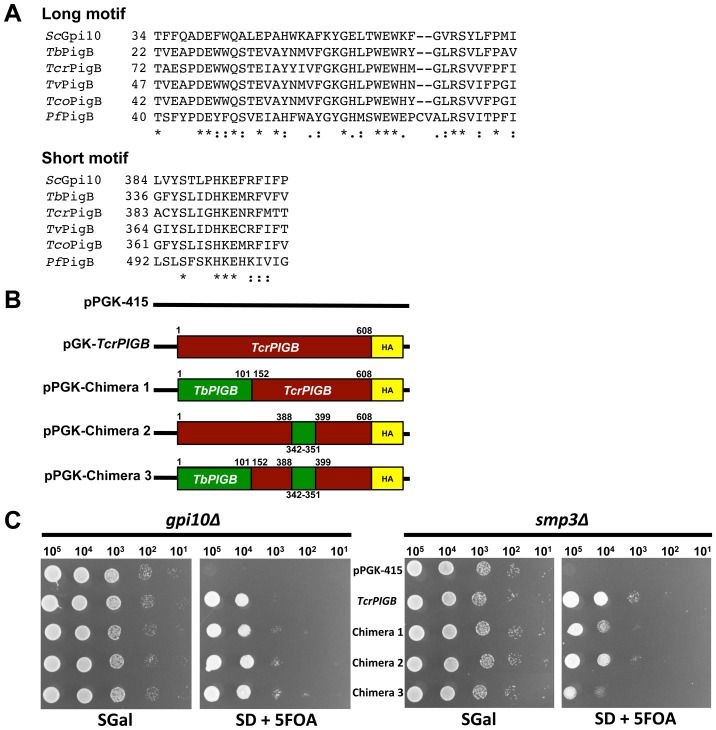
The long and short conserved motifs do not confer broad specificity to *Tcr*PigB. A. Alignment of the N-terminal long and C-terminal short motifs from yeast and protozoa. B. Schematics of the generated chimera constructs. The N-terminal long (chimera 1), C-terminal short (chimera 2) or both conserved motifs (chimera 3) of *TcrPIGB* (red) were replaced by those from *TbPIGB* (green). Numbers correspond to the amino acid positions of each domain within its native protein sequence. C. Serial drop cultures of control and chimeric constructs in both *gpi10*Δ and *smp3*Δ strains before (SGal) and after (SD + 5FOA) plasmid shuffling. Note that all three of the chimeric PigB proteins are able to complement the *gpi10*Δ and *smp3*Δ mutant strains.

Each of the sequences of the parasite PigB proteins used in this study contained a conserved N-terminal “long motif” and a C-terminal HKEXRF “short motif” (Figure 5A) that were previously defined in bioinformatics studies that examined GPI mannosyltransferase sequences [32], [34]. These domains reside within putative loop regions of the protein and are hypothesized to be a part of the active site (long motif) or substrate acceptor site (short motif) of the enzyme [32]. We wished to determine whether either of these motifs was important for the ability of some parasite PigB proteins to add both Man-III and Man-IV to GPI precursors. Chimeric proteins were designed where the region of the protein containing the long motif and/or the short motif of the broader specificity *Tcr*PigB protein was replaced by the protein domain containing the appropriate long (chimera 1) and/or short (chimeras 2 and 3) motifs from *Tb*PigBp (Figure 5B). We selected *TbPIGB* and *TcrPIGB* for these experiments because they were from closely related organisms and they both gave robust complementation of *gpi10*Δ (*TbPIGB* and *TcrPIGB*) and/or *smp3*Δ (only *TcrPIGB*).

Vectors directing expression of each chimera were introduced into haploid *gpi10*Δ and *smp3*Δ strains and were subjected to plasmid shuffling as described above. All three chimeric proteins were able to compensate for defects in Man-III addition to GPI precursors, indicating they were functional proteins (Figure 5C). Interestingly, despite containing the long and/or short motif sequences from the exclusively Man-III mannosyltransferase *TbPIGB*, each of the *TcrPIGB* chimeras was also still able to compensate for a deficiency in Man-IV addition to GPI precursors (Figure 5C). These data suggest that the two most highly conserved regions of PigB proteins are not alone or in combination responsible for the ability of some PigB proteins to discriminate between Man_2_-GPI and Man_3_-GPIs substrates for mannose transfer.

## Discussion

In this study, after an exhaustive bioinformatics search, we found no *SMP3/PIGZ* sequences in the genomes of various protozoan parasites, despite prior reports that *P. falciparum* and *T. cruzi* harbor Man-IV on the GPI anchors of several GPI anchored proteins [16], [17]. We hypothesized that the α(1,2)-mannosyltransferase responsible for Man-III addition (PigB) might also be capable of adding Man-IV in these organisms. Because addition of Man-III and Man-IV to GPI precursors is performed by two separate essential mannosyltransferases in *S. cerevisiae*, we utilized complementation of yeast null mutants to explore the *in vivo* specificities of proteins encoded by various parasite PigB proteins. We showed that all parasite *PIGB* genes we examined could be functionally expressed in yeast and restored viability to cells defective in essential GPI-MT-III activity due to deletion of *gpi10*. Additionally, we found that expression of the *PIGB* genes from *T. cruzi*, *T. congolense* and *P. falciparum* restored viability to cells defective in essential GPI-MT-IV activity (*smp3*Δ. Finally, in a definitive experiment, we showed that *T. cruzi PIGB* was able to robustly complement a yeast *gpi10*Δ/*smp3*Δ double mutant strain defective in addition of both essential α(1,2)-linked mannoses (Man-III and Man-IV) to GPI precursors.

The likeliest interpretation of our complementation data is that certain parasite *PIGB* genes encode GPI mannosyltransferases that are capable of adding two α(1,2)-linked mannoses (Man-III and Man-IV) to GPI intermediate lipids during their biosynthesis *in vivo*. Interestingly, the pattern of complementation we observed correlated to the known presence or absence of Man-IV on GPI anchored proteins isolated from individual parasites. For example, *TcrPIGB* and *PfPIGB* each showed broad specificity by complementing both *gpi10*Δ and *smp3*Δ in yeast and Man_4_-GPI structures have been reported on GPI anchored proteins in both *T. cruzi* and *P. falciparum* [16], [17]. In contrast, *TbPIGB* complemented only *gpi10*Δ and Man-IV is not present on the two characterized GPIs from the VSG [20] and procyclin [35] proteins of *T. brucei*. Thus, broad specificity PigB proteins represent a possible mechanism by which Man_4_-GPIs are formed by protozoan parasites lacking *SMP3/PIGZ*. Furthermore, the absence of an *SMP3/PIGZ* sequence encoded in any eukaryotic genome likely does not exclusively predict the organism's inability to modify its GPIs with Man-IV.

In conclusion, our study represents the first report implicating a GPI mannosyltransferase in performing multiple sugar additions to the glycan of GPI intermediate lipids. Furthermore, the difference in specificity of some protozoan parasite PigB proteins and those of higher eukaryotes suggests that selective inhibition of PigBp-mediated mannose transfer to GPIs may be a plausible strategy for development of novel anti-parasitic chemotherapeutic agents.

## Supporting Information

Table S1 Putative Gpi10/PigB and Smp3/PigZ proteins present in Kingdom Animalia.
(DOC)

Table S2 Primers used in the generation of plasmids.
(DOC)

Table S3 Primers used to confirm yeast strains.
(DOC)
