# Supplementary material for: Complementation of Essential Yeast GPI Mannosyltransferase Mutations Suggests a Novel Specificity for Certain Trypanosoma and Plasmodium PigB Proteins
Source: PLoS One. 2014 Jan 29;9(1):e87673. doi: 10.1371/journal.pone.0087673 (PMC3906172; doi:10.1371/journal.pone.0087673)
Supplement: Table S1 — Putative Gpi10/PigB and Smp3/PigZ proteins present in Kingdom Animalia. (DOC) [file pone.0087673.s001.doc]

**Table S1. Putative Gpi10/PigB and Smp3/PigZ proteins present in Kingdom Animalia**

|  |  | | |  | **Gpi10/PigB** | |  | **Smp3/PIGZ** | |
| --- | --- | --- | --- | --- | --- | --- | --- | --- | --- |
|  | **Organism** | | | **Common Name or Organism Type** | **GenBank**  **Accession** | **Protein**  **Length*^a^*** |  | **GenBank**  **Accession** | **Protein**  **Length*^a^*** |
| **I. Vertebrates** | | | |  |  |  |  |  |  |
|  | **Amphibians** | | |  |  |  |  |  |  |
|  | *Xenopus laevis* | | | frog | NP_001089512 | 531 |  | not found | - |
|  | *Xenopus tropicalis* | | | frog | NP_001120608 | 528 |  | XP_002937887 | 563 |
|  |  | | |  |  |  |  |  |  |
|  | **Birds** | | |  |  |  |  |  |  |
|  | *Anas platyrhynchos* | | | duck | EOB06917 | 445 |  | EOB02296 | 515 |
|  | *Columba livia* | | | pigeon | XP_005512327 | 467 |  | XP_005516090 | 526 |
|  | *Falco peregrinus* | | | falcon | XP_005241545 | 562 |  | XP_005237847 | 559 |
|  | *Ficedula albicollis* | | | flycatcher | XP_005052033 | 425 |  | XP_005051115 | 556 |
|  | *Gallus gallus* | | | chicken | XP_003641928 | 705 |  | XP_426699 | 559 |
|  | *Geospiza fortis* | | | finch | XP_005419720 | 425 |  | XP_005428058 | 534 |
|  | *Meleagris gallopavo* | | | turkey | XP_003209484 | 439 |  | XP_003209152 | 580 |
|  | *Melopsittacus undulatus* | | | budgerigar | XP_005145991 | 796 |  | XP_005147074 | 559 |
|  | *Pseudopodoces humilis* | | | ground-tit | XP_005521534 | 452 |  | XP_005527139 | 558 |
|  | *Taeniopygia guttata* | | | zebra finch | XP_002195841 | 598 |  | XP_002188983 | 650 |
|  | *Zonotrichia albicollis* | | | sparrow | XP_005487904 | 520 |  | XP_005489494 | 522 |
|  |  | | |  |  |  |  |  |  |
|  | **Fish** | | |  |  |  |  |  |  |
|  | *Danio rerio* | | | zebrafish | NP_956770 | 536 |  | not found | - |
|  | *Maylandia zebra* | | | Zebra Mbuna | XP_004543574 | 450 |  | not found | - |
|  | *Oreochromis niloticus* | | | tilapia | XP_003442392 | 524 |  | not found | - |
|  | *Oryzias latipes* | | | medaka | XP_004067233 | 524 |  | not found | - |
|  | *Takifugu rubripes* | | | fugu | XP_003970094 | 550 |  | not found | - |
|  | *Tetraodon nigroviridis* | | | pufferfish | CAF99570 | 525 |  | not found | - |
|  |  | | |  |  |  |  |  |  |
|  | **Mammals** | | |  |  |  |  |  |  |
|  | *Ailuropoda melanoleuca* | | | panda | XP_002922997 | 666 |  | XP_002916819 | 600 |
|  | *Bos grunniens mutus* | | | yak | ELR49159 | 523 |  | ELR54922 | 574 |
|  | *Bos taurus* | | | cow | NP_001071523 | 541 |  | XP_005196147 | 563 |
|  | *Callithrix jacchus* | | | marmoset | XP_002753514 | 322 |  | XP_003734869 | 576 |
|  | *Canis lupus familiaris* | | | dog | XP_005638746 | 541 |  | XP_005639667 | 598 |
|  | *Cavia porcellus* | | | rodent | XP_003471839 | 942 |  | XP_005006944 | 575 |
|  | *Ceratotherium simum simum* | | | rhinoceros | XP_004421662 | 541 |  | XP_004424857 | 592 |
|  | *Chinchilla lanigera* | | | rodent | XP_005377194 | 411 |  | XP_005383208 | 633 |
|  | *Condylura cristata* | | | mole | XP_004687586 | 639 |  | XP_004675478 | 583 |
|  | *Cricetulus griseus* | | | hamster | XP_003495071 | 547 |  | EGV96364 | 565 |
|  | *Dasypus novemcinctus* | | | armadillo | XP_004468381 | 540 |  | XP_004465949 | 567 |
|  | *Echinops telfairi* | | | hedgehog | XP_004709578 | 540 |  | XP_004712392 | 403 |
|  | *Equus caballus* | | | horse | XP_001501050 | 541 |  | XP_001916639 | 592 |
|  | *Felis catus* | | | cat | XP_003987178 | 541 |  | XP_003991824 | 592 |
|  | *Gorilla gorilla* | | | gorilla | XP_004056275 | 539 |  | XP_004038318 | 579 |
|  | *Heterocephalus glaber* | | | rodent | XP_004835407 | 539 |  | XP_004834775 | 571 |
|  | *Homo sapiens* | | | human | NP_004846 | 554 |  | NP_079439 | 579 |
|  | *Ictidomys tridecemlineatus* | | | squirrel | XP_005316650 | 544 |  | XP_005341033 | 642 |
|  | *Jaculus jaculus* | | | rodent | XP_004662478 | 677 |  | XP_004654600 | 577 |
|  | *Loxodonta africana* | | | elephant | XP_003418431 | 541 |  | XP_003412851 | 530 |
|  | *Macaca fascicularis* | | | monkey | EHH63097 | 556 |  | XP_005545366 | 579 |
|  | *Macaca mulatta* | | | monkey | XP_002804830 | 551 |  | EHH16877 | 579 |
|  | *Mesocricetus auratus* | | | rodent | XP_005073798 | 547 |  | XP_005071622 | 560 |
|  | *Microtus ochrogaster* | | | rodent | XP_005347694 | 550 |  | XP_005344883 | 559 |
|  | *Mus musculus* | | | mouse | NP_061377 | 542 |  | NP_766410 | 560 |
|  | *Mustela putorius furo* | | | ferret | XP_004795953 | 542 |  | XP_004745562 | 616 |
|  | *Myotis brandtii* | | | bat | EPQ03932 | 501 |  | EPQ12033 | 568 |
|  | *Myotis davidii* | | | bat | XP_004639098 | 551 |  | ELK38031 | 568 |
|  | *Nomascus leucogenys* | | | gibbon | XP_003266999 | 554 |  | XP_003280168 | 579 |
|  | *Ochotona princeps* | | | pika | XP_004578422 | 575 |  | XP_004578303 | 598 |
|  | *Octodon degus* | | | rodent | XP_004639098 | 551 |  | XP_004644627 | 567 |
|  | *Odobenus rosmarus* | | | walrus | XP_004404021 | 541 |  | XP_004392030 | 580 |
|  | *Orcinus orca* | | | killer whale | XP_004274806 | 541 |  | XP_004278882 | 592 |
|  | *Ornithorhynchus anatinus* | | | platypus | XP_001514392 | 611 |  | XP_001512679 | 716 |
|  | *Oryctolagus cuniculus* | | | rabbit | XP_002717759 | 541 |  | XP_002716443 | 561 |
|  | *Ovis aries* | | | sheep | XP_004011061 | 530 |  | XP_004003862 | 591 |
|  | *Pan paniscus* | | | chimpanzee | XP_003827909 | 554 |  | XP_003806471 | 579 |
|  | *Pan troglodytes* | | | chimpanzee | XP_003314729 | 554 |  | XP_003950308 | 502 |
|  | *Papio anubis* | | | baboon | XP_003901022 | 542 |  | XP_003895367 | 579 |
|  | *Pongo abelii* | | | orangutan | XP_002825522 | 536 |  | XP_002814516 | 409 |
|  | *Rattus norvegicus* | | | rat | NP_001101636 | 542 |  | XP_002727945 | 558 |
|  | *Saimiri boliviensis* | | | monkey | XP_003935646 | 535 |  | XP_003926246 | 548 |
|  | *Sorex araneus* | | | shrew | XP_004602026 | 768 |  | XP_004603293 | 633 |
|  | *Sus scrofa* | | | pig | XP_005659643 | 542 |  | XP_003132653 | 589 |
|  | *Trichechus manatus* | | | manatee | XP_004374831 | 542 |  | XP_004373763 | 590 |
|  | *Tursiops truncatus* | | | dolphin | XP_004317938 | 541 |  | XP_004321658 | 592 |
|  |  | | |  |  |  |  |  |  |
| **II. Invertebrates** | | | |  |  |  |  |  |  |
|  | **Arthropods** | | |  |  |  |  |  |  |
|  | *Acromyrmex echinatior* | | | ant | EGI64807 | 499 |  | EGI62118 | 680 |
|  | *Acyrthosiphon pisum* | | | aphid | XP_001949355 | 485 |  | XP_003246197 | 592 |
|  | *Aedes aegypti* | | | mosquito | XP_001647883 | 517 |  | XP_001651650 | 683 |
|  | *Anopheles gambiae* | | | mosquito | XP_310348 | 519 |  | XP_309785 | 680 |
|  | *Apis mellifera* | | | honey bee | XP_397080 | 1215 |  | XP_624036 | 674 |
|  | *Bombyx mori* | | | silkworm | XP_004923797 | 506 |  | XP_004923921 | 637 |
|  | *Camponotus floridanus* | | | ant | EFN69242 | 499 |  | EFN70092 | 667 |
|  | *Ceratitis capitata* | | | fruit fly | XP_004525173 | 565 |  | XP_004536849 | 683 |
|  | *Culex quinquefasciatus* | | | mosquito | XP_001864746 | 519 |  | XP_001844944 | 678 |
|  | *Danaus plexippus* | | | monarch butterfly | EHJ64801 | 402 |  | EHJ64742 | 654 |
|  | *Daphnia pulex* | | | water flee | EFX79028 | 475 |  | EFX84270 | 612 |
|  | *Dendroctonus ponderosae* | | | beetle | ERL86863 | 457 |  | ENN70166 | 362 |
|  | *Drosophila ananassae* | | | fly | XP_001957108 | 523 |  | XP_001960928 | 696 |
|  | *Drosophila erecta* | | | fly | XP_001971786 | 531 |  | XP_001976590 | 826 |
|  | *Drosophila grimshawi* | | | fly | XP_001984385 | 522 |  | XP_001985775 | 708 |
|  | *Drosophila melanogaster^d^* | | | fly | NP_995991 | 531 |  | NP_995937 | 696 |
|  | *Drosophila mojavensis* | | | fly | XP_002007203 | 518 |  | XP_002005737 | 704 |
|  | *Drosophila persimilis* | | | fly | XP_002026997 | 756 |  | XP_002026361 | 707 |
|  | *Drosophila pseudoobscura* | | | fly | XP_001353683 | 519 |  | XP_002138987 | 707 |
|  | *Drosophila sechellia* | | | fly | XP_002035230 | 531 |  | XP_002043049 | 818 |
|  | *Drosophila simulans* | | | fly | XP_002083521 | 555 |  | XP_002082952 | 689 |
|  | *Drosophila virilis* | | | fly | XP_002046563 | 519 |  | XP_002049460 | 707 |
|  | *Drosophila willistoni* | | | fly | XP_002068542 | 521 |  | XP_002062889 | 703 |
|  | *Drosophila yakuba* | | | fly | XP_002093587 | 527 |  | XP_002092810 | 696 |
|  | *Harpegnathos saltator* | | | ant | EFN78076 | 658 |  | EFN88229 | 693 |
|  | *Ixodes scapularis* | | | black-legged tick | XP_002409576 | 490 |  | XP_002400895 | 470 |
|  | *Megachile rotundata* | | | bee | XP_003703935 | 1212 |  | XP_003707649 | 675 |
|  | *Pediculus humanus corporis* | | | human body louse | XP_002429777 | 425 |  | XP_002425368 | 754 |
|  | *Nasonia vitripennis* | | | jewel wasp | XP_001600895 | 941 |  | XP_001603565 | 667 |
|  | *Solenopsis invicta* | | | red fire ant | EFZ09140 | 526 |  | EFZ12284 | 681 |
|  | *Tribolium castaneum* | | | red flour beetle | NP_001161228 | 491 |  | XP_971862 | 638 |
|  |  | | |  |  |  |  |  |  |
|  | **Primitive multicellular animals** | | |  |  |  |  |  |  |
|  | *Amphimedon queenslandica* | | | sponge | XP_003387730 | 546 |  | not found | - |
|  | *Aplysia californica* | | | sea hare | XP_005092156 | 647 |  | XP_005092851 | 637 |
|  | *Branchiostoma floridae* | | | Florida lancet | XP_002601540 | 340 |  | XP_002603432 | 582 |
|  | *Brugia malayi* | | | round worm | XP_001892406 | 612 |  | not found | - |
|  | *Caenorhabditis brenneri* | | | round worm | EGT40899 | 496 |  | not found | - |
|  | *Caenorhabditis briggsae* | | | round worm | XP_002630627 | 495 |  | not found | - |
|  | *Caenorhabditis elegans* | | | round worm | NP_001254097 | 496 |  | not found | - |
|  | *Caenorhabditis remanei* | | | round worm | XP_003109012 | 496 |  | not found | - |
|  | *Capitella teleta* | | | worm | ELU03730 | 497 |  | ELT97792 | 478 |
|  | *Ciona intestinalis* | | | sea squirt | XP_004227268 | 539 |  | XP_002125899 | 618 |
|  | *Clonorchis sinensis* | | | liver fluke | GAA55710 | 565 |  | GAA49133 | 835 |
|  | *Hydra magnipapillata* | | | hydrozoan | XP_002159729 | 1049 |  | XP_002168230 | 409 |
|  | *Loa loa* | | | eye worm | XP_003145696 | 587 |  | not found | - |
|  | *Nematostella vectensis* | | | sea anemone | XP_001637772 | 491 |  | XP_001634575 | 452 |
|  | *Saccoglossus kowalevskii* | | | worm | XP_002736845 | 375 |  | XP_002730481 | 515 |
|  | *Schistosoma mansoni* | | | flatworm | XP_002576640 | 570 |  | XP_002573748 | 1648 |
|  | *Strongylocentrotus purpuratus* | | | sea urchin | XP_788180 | 568 |  | XP_784333 | 616 |
|  | *Wuchereria bancrofti* | | | round worm | EJW82632 | 581 |  | not found | - |
|  |  | | |  |  |  |  |  |  |
|  | **Protozoa** | | |  |  |  |  |  |  |
|  | *Angomonas deanei* | | | parasite | EPY18195 | 569 |  | not found | - |
|  | *Capsaspora owczarzaki* | | | amoeba | XP_004364424 | 517 |  | EFW45898 | 536 |
|  | *Cryptosporidium hominis* | | | parasite | XP_666282 | 626 |  | not found | - |
|  | *Cryptosporidium muris* | | | parasite | XP_002140238 | 600 |  | not found | - |
|  | *Cryptosporidium parvum* | | | parasite | XP_626909 | 637 |  | not found | - |
|  | *Dictyostelium discoideum* | | | slime mold | XP_638629 | 547 |  | XP_001134503 | 667 |
|  | *Dictyostelium fasciculatum* | | | slime mold | XP_004361080 | 511 |  | XP_004354958 | 516 |
|  | *Ichthyophthirius multifiliis* | | | parasite | XP_004034737 | 486 |  | not found | - |
|  | *Leishmania infantum* | | | parasite | XP_001469483 | 626 |  | not found | - |
|  | *Naegleria gruberi* | | | amoeboflagellate | XP_002674306 | 284 |  | XP_002683610 | 554 |
|  | *Paramecium tetraurelia* | | | ciliate | XP_001461426 | 485 |  | not found | - |
|  | *Perkinsus marinus* | | | alveolate | XP_002787691 | 522 |  | not found | - |
|  | *Phytophthora infestans* | | | oomycete | XP_002997733 | 528 |  | XP_002903345 | 567 |
|  | *Phytophthora sojae* | | | oomycete | EGZ27706 | 533 |  | EGZ13881 | 585 |
|  | *Plasmodium berghei* | | | parasite | XP_677099 | 391 |  | not found | - |
|  | *Plasmodium chabaudi* | | | parasite | XP_744108 | 624 |  | not found | - |
|  | *Plasmodium falciparum* | | | parasite | XP_001350137 | 786 |  | not found | - |
|  | *Plasmodium knowlesi* | | | parasite | XP_002259939 | 738 |  | not found | - |
|  | *Plasmodium yoelii* | | | parasite | XP_729140 | 240 |  | not found | - |
|  | *Polysphondylium pallidum* | | | slime mold | EFA77662 | 763 |  | EFA77662 | 763 |
|  | *Saprolegnia diclina* | | | cotton mold | EQC29704 | 499 |  | EQC38134 | 525 |
|  | *Tetrahymena thermophila* | | | ciliate | XP_001014583 | 552 |  | not found | - |
|  | *Toxoplasma gondii* | | | parasite | CAJ20693 | 768 |  | not found | - |
|  | *Trichoplax adhaerens* | | | placozoa | XP_002110590 | 356 |  | XP_002110340 | 503 |
|  | *Trypanosoma brucei* | | | parasite | BAA94863 | 558 |  | not found | - |
|  | *Trypanosoma congolense* | | | parasite | CCC93706 | 583 |  | not found | - |
|  | *Trypanosoma cruzi* | | | parasite | XP_806762 | 608 |  | not found | - |
|  | *Trypanosoma vivax* | | | parasite | CCC51511 | 588 |  | not found | - |
|  |  | | |  |  |  |  |  |  |
| *^a^*Cited protein lengths are as indicated by each protein’s GenBank sequence annotation. No attempts were made to verify the accuracy of each reported protein sequence. | | | | | | | | | |
|  |  |  |  | |  | | | | |
