# Supplementary material for: Complementation of Essential Yeast GPI Mannosyltransferase Mutations Suggests a Novel Specificity for Certain Trypanosoma and Plasmodium PigB Proteins
Source: PLoS One. 2014 Jan 29;9(1):e87673. doi: 10.1371/journal.pone.0087673 (PMC3906172; doi:10.1371/journal.pone.0087673)
Supplement: Table S2 — Primers used in the generation of plasmids. (DOC) [file pone.0087673.s002.doc]

**Table S2. Primers used in the generation of plasmids**

| **Primer** | **Target** | **Sequence** |
| --- | --- | --- |
| 1 | 5'end *TcrPIGB* | 5’-ATAAGAATGCGGCCGCAAAATGTCTCCTTGTATTGTGTATTGTTTC-3’ |
| 2 | 3'end *TcrPIGB* with HA tag | 5’-TTAGGCGCGCCTTATGCATAATCTGGAACATCATATGGATAATTGGCCTGAACAACATTT CGTACCCA-3’ |
| 3 | 5'end *TbPIGB* | 5’-ATAAGAATGCGGCCGCAAAATGCCGTGGTGGTTGATTTCTCTC-3’ |
| 4 | 3'end *TbPIGB* with HA tag | 5’-TTAGGCGCGCCTTATGCATAATCTGGAACATCATATGGATACTTCGTCACCTTGACCCAC ATTTG-3’ |
| 5 | 5'end *TvPIGB* | 5’-ATAAGAATGCGGCCGCATGCCCGGTAGCACAGTGCCGGTA-3’ |
| 6 | 3'end *TvPIGB* with HA tag | 5’-TTAGGCGCGCCTTATGCATAATCTGGAACATCATATGGATAGTTGTCGACAGCCCGGAC CCATAACTC-3’ |
| 7 | 5'end *TcoPIGB* | 5’-ATAAGAATGCGGCCGCATGATGGGCAGTAAAGAAGTTAGAGCA-3’ |
| 8 | 3'end *TcoPIGB* with HA tag | 5’-TTAGGCGCGCCTTATGCATAATCTGGAACATCATATGGATAGGTGTTCATTTTGCTCCAC ATTTCAAT-3’ |
| 9 | 5'end *PfPIGB*^1^ | 5’-ATAAGAATGCGGCCGCATGATCTATAACGATATCTTGACATTGTGTGC-3’ |
| 10 | 3'end *PfPIGB*^1^ with HA tag | 5’-TTAGGCGCGCCTTATGCATAATCTGGAACATCATATGGATAAGAAGGAACTCTCTTAAAA ATATAATGGTAAACTGG-3’ |
| 11 | 5'end *ScSMP3* | 5’-GATTGCGGCCGCATGATGAGGTATCAATGGTGGC-3’ |
| 12 | 3'end *ScSMP3* | 5’-AAGGCGCGCCTTATAGTAGTTCGATGGAGTACACTG-3’ |
| 13 | 5'end *ScGPI10* | 5’-CATGCTGCAGATGGCTCACGAGGTTCATAGAAT-3’ |
| 14 | 3'end *ScGPI10* | 5’-CATGGATCCTTAAATATCTGCCGCTGGAATATCGC-3’ |
| 15 | *TbPIGB* C-terminal conserved domain into *TcrPIGB* for | 5’-GGATTCTATTCCTTGATTGATCACAAAGAAATGCGTTTTGTTTTTGTAGTGTTGCCAATTT GTTTTGTGGTGACAGCC-3’ |
| 16 | *TbPIGB* C-terminal conserved domain into *TcrPIGB* rev | 5’-TACAAAAACAAAACGCATTTCTTTGTGATCAATCAAGGAATAGAATCCAACGGGCCATAA AATAAAAAAGAACAATCC-3’ |

^1^*PfPIGB* was codon optimized for yeast expression
