# Supplementary material for: Complementation of Essential Yeast GPI Mannosyltransferase Mutations Suggests a Novel Specificity for Certain Trypanosoma and Plasmodium PigB Proteins
Source: PLoS One. 2014 Jan 29;9(1):e87673. doi: 10.1371/journal.pone.0087673 (PMC3906172; doi:10.1371/journal.pone.0087673)
Supplement: Table S3 — Primers used to confirm yeast strains. (DOC) [file pone.0087673.s003.doc]

**Table S3. Primers used to confirm yeast strains**

| **Primer** | **Target** | **Sequence** |
| --- | --- | --- |
| P1 | *URA3* | 5'-CGTGCTGCTACTCATCCTAG-3' |
| P2 | *URA3* | 5'-GTCGCTCTTCGCAATGTCAAC-3' |
| P3 | 5'UTR *ScGPI10* | 5'-TTTAAGTCAGAATCCTTTGAAATGCTGC-3' |
| P4 | Kanamycin | 5'-CTGCAGCGAGGAGCCGTAAT-3' |
| P5 | 5'UTR *ScSMP3* | 5'-GCGGACTGTAATGTTTGTAAGAAGT-3' |
| P6 | pGK-415 | 5'-CACACAGGAAACAGCTATGACCATG-3' |
| P7 | *ScGPI10* | 5'-CATTCGTGTCGGTGTTCATCGCAT-3' |
| P8 | pGK-415 | 5'-CGACTCACTATAGGGCGAATTGG-3' |
| P9 | *ScSMP3* | 5'-CTTACGCAATCATATCTGGCCTGC-3' |
| P10 | *TbPIGB* | 5'-GAACCGTCTGTTTGTATTGTTCAAC-3' |
| P11 | *TcrPIGB* | 5'-CACGTGTCATACGTCGCCTTCAT-3' |
| P12 | *TvPIGB* | 5'-CTCACTTATTGACCACAAGGAATGC-3' |
| P13 | *TcoPIGB* | 5'-CTAGGTATTGCTGCATGGACGGTG-3' |
| P14 | *PfPIGB^1^* | 5'-GATCTCCTGAACATGTTGCTTCATACTTC-3' |

^1^*PfPIGB* was codon optimized for yeast expression
